# Troponin I Assay for Identification of a Significant Coronary Stenosis in Patients with Suspected Acute Myocardial Infarction and Wide QRS Complex

**DOI:** 10.1371/journal.pone.0154724

**Published:** 2016-05-05

**Authors:** Beatrice von Jeinsen, Stergios Tzikas, Gerhard Pioro, Lars Palapies, Tanja Zeller, Christoph Bickel, Karl J. Lackner, Stephan Baldus, Stefan Blankenberg, Thomas Muenzel, Andreas M. Zeiher, Till Keller

**Affiliations:** 1 Division of Cardiology, Department of Internal Medicine III, Goethe University Frankfurt, Frankfurt, Germany; 2 3rd Department of Cardiology, Aristotle University of Thessaloniki, Ippokrateio Hospital, Thessaloniki, Greece; 3 Department of General and Interventional Cardiology, University Heart Center, Hamburg, Germany; 4 Department of Internal Medicine, Federal Armed Forces Hospital, Koblenz, Germany; 5 Department of Laboratory Medicine, University Medical Center, Johannes Gutenberg University, Mainz, Germany; 6 Department of Internal Medicine III, University of Cologne, Köln, Germany; 7 Department of Internal Medicine II, University Medical Center, Johannes Gutenberg University, Mainz, Germany; 8 German Centre for Cardiovascular Research (DZHK), Berlin, Germany; University of Bologna, ITALY

## Abstract

**Background:**

Common ECG criteria such as ST-segment changes are of limited value in patients with suspected acute myocardial infarction (AMI) and bundle branch block or wide QRS complex. A large proportion of these patients do not suffer from an AMI, whereas those with ST-elevation myocardial infarction (STEMI) equivalent AMI benefit from an aggressive treatment. Aim of the present study was to evaluate the diagnostic information of cardiac troponin I (cTnI) in hemodynamically stable patients with wide QRS complex and suspected AMI.

**Methods:**

In 417 out of 1818 patients presenting consecutively between 01/2007 and 12/2008 in a prospective multicenter observational study with suspected AMI a prolonged QRS duration was observed. Of these, n = 117 showed significant obstructive coronary artery disease (CAD) used as diagnostic outcome variable. cTnI was determined at admission.

**Results:**

Patients with significant CAD had higher cTnI levels compared to individuals without (median 250ng/L vs. 11ng/L; p<0.01). To identify patients needing a coronary intervention, cTnI yielded an area under the receiver operator characteristics curve of 0.849. Optimized cut-offs with respect to a sensitivity driven *rule-out* and specificity driven *rule-in* strategy were established (40ng/L/96ng/L). Application of the specificity optimized cut-off value led to a positive predictive value of 71% compared to 59% if using the 99^th^ percentile cut-off. The sensitivity optimized cut-off value was associated with a negative predictive value of 93% compared to 89% provided by application of the 99^th^ percentile threshold.

**Conclusion:**

cTnI determined in hemodynamically stable patients with suspected AMI and wide QRS complex using optimized diagnostic thresholds improves *rule-in* and *rule-out* with respect to presence of a significant obstructive CAD.

## Introduction

In patients presenting to an emergency room (ER) with acute chest pain, the electrocardiogram (ECG) is a major cornerstone for timely diagnose of an acute myocardial infarction (AMI) based on the presence of ST-elevations. Patients presenting with an ST-elevation myocardial infarction (STEMI) require aggressive and immediate further treatment including an early percutaneous coronary intervention (PCI) or fibrinolytic therapy according to current guidelines[1,2].

Patients suffering from acute chest pain, presenting without electrocardiographic ST elevations, undergo serial biomarker testing, preferably cardiac troponin I/T, to diagnose or to rule out a non-ST-elevation myocardial infarction (NSTEMI)[3]. In these patients, an early interventional strategy is not always the preferred therapeutic approach[4].

In case of the presence of a left bundle branch block (LBBB), the standard ECG-criteria for STEMI are of limited use due to the repolarization disorder in the presence of LBBB. According to former guidelines of the European Society of Cardiology (ESC) and the American Heart Association (AHA), patients presenting with acute chest pain, whose ECG shows an assumingly new LBBB should be treated as STEMI with immediate emergency coronary angiography / fibrinolytic therapy [2,5]. According to the latest revision of these guidelines, this section has been revised and patients suffering from acute chest pain presenting with new or presumably new LBBB are no longer viewed as STEMI patients in general[1].

Different attempts have been proposed to help identifying an AMI in the presence of LBBB. Sgarbossa et al. proposed an algorithm, in which the extent of the ST-elevation and/or depression is used as electrocardiographic criteria to identify AMI[6]. This approach has been discussed controversially[7,8,9,10]. Additionally, modification of the Sgarbossa criteria and other ECG criteria have been suggested to further improve the identification of patients suffering from AMI presenting with LBBB[11,12,13].

Furthermore, the need of an emergency PCI / fibrinolytic therapy in all patients with presumed newly diagnosed LBBB is a matter of debate. Several authors state that these patients should be treated like patients suffering from STEMI [14,15,16] and should undergo an emergency PCI [17], whereas others claim that most of these patients are not suffering from AMI and an emergency reperfusion therapy would lead to a higher mortality [18,19,20,21,22]. Moreover, newly diagnosed Right Bundle Branch Block (RBBB) in patients presenting with acute chest pain and prolonged QRS time is also associated with higher 30 day mortality[23].

Taking these findings together underlines the difficulties in diagnosis and treatment decision in patients presenting with acute chest pain and LBBB to an ER.

Aim of the present investigation is to evaluate the predictive information of sensitively determined cardiac troponin I (cTnI) in hemodynamically stable patients, presenting with wide QRS complex and suspected AMI, with respect to the presence of a significant obstructive coronary artery disease (CAD) and, therefore, to identify patients who might benefit from an early coronary intervention.

## Material and Methods

### Study population

The present study was approved by the local ethics committees of Rheinland-Pfalz and Hamburg. Participation was voluntary and each patient gave written informed consent.

Between January 2007 and December 2008, a total of 1,818 patients presented consecutively to the chest pain units of 3 German study centers with AMI and participated in the present study, as previously published[24]. Valid data on QRS duration on admission was available in 1790 patients. Of these, 427 patients showed at least a partial bundle branch block based on a prolonged QRS duration of at least 110ms and were selected for further analysis. 246 Patients had a QRS duration of at least 120ms and 204 presented with LBBB.

All patients older than 18 years and younger than 85 years of age with symptoms suggestive of an acute coronary syndrome were eligible to participate. Exclusion criteria were trauma or major surgery within the last 4 weeks, pregnancy, intrave- nous drug abuse, and anemia. Data on cardiovascular risk factors was obtained as described earlier[25]. A total of 820 patients underwent coronary angiography, 56% within 1 day.

### Electrocardiographic assessment

Directly upon admission, a 12-lead ECG was obtained under standardized conditions in the respective chest pain observation unit used for clinical decision making by the treating physician and stored for further analyses.

For the present evaluation, ECGs were post-hoc analyzed by scientific staff blinded to the patients’ characteristics or diagnoses. Presence of left or right bundle-branch block was classified according to standard ECG criteria[26]. QRS duration was measured using built in algorithms of the used ECG device. To ensure comparability, n = 50 ECGs were randomly chosen, in which the QRS time was measured manually in all 12 leads, and their mean was compared to the QRS duration measured in the Einthoven II lead with a resulting coefficient of variation of 4.28%. Additionally, 50 different ECGs were randomly chosen and the manually measured QRS duration using the Einthoven II lead was compared to the device calculated QRS duration with a coefficient of variation of 4.37%. A prolonged QRS time was defined as duration of at least 110ms.

Out of 246 patients with a QRS time of at least 120ms an ECG analysis according to the Sgarbossa Algorithm was carried out. Analysis was possible in 206 patients, 40 ECGs were not available for further analysis. According to the Sgarbossa algorithm, we screened for ST-segment elevation ≥1 mm concordant with QRS complex and weighted a positive result with 5 points, screened for ST-segment depression ≥1 mm in lead V1, V2, or V3 and weighted a positive result with 3 points and screened for ST-segment elevation ≥5 mm discordant with QRS complex and weighted a positive result with 2 points. We calculated the Index Score according to the Sgarbossa algorithm by adding the respective points; If the Index Score achieved a result of at least 3 points, the respective ECG was considered as a predictor of an AMI. Respective Sensitivity, Specificity, negativ and positive predicitive values were derived [6].

### Definition of the final diagnosis

Since cardiac troponin levels as well as the presence of a wide QRS complex with presumably new bundle branch block configuration is part of the definition of myocardial infarction, we did not use myocardial infarction as the diagnosis to identify. Instead, we choose the need for a coronary intervention as the diagnosis to identify based on the assumption, that patients that benefit the most from an early invasive treatment are the ones requiring revascularization therapy in the presence of wide QRS complex. Need for coronary intervention was defined as a culprit lesion according to visual criteria as the Ambrose criteria was present and a percutaneous coronary intervention (balloon angioplasty and/or stent implantation; n = 105) was performed and/or patients were referred for coronary artery bypass graft surgery (n = 15) based on the decision of the treating cardiologists.

Of the 427 patients with wide QRS complex, a total of 118 patients (2 patients had a coronary artery bypass surgery after percutaneous coronary intervention) underwent coronary intervention. Of the 246 patients with QRS duration of at least 120ms 60 patients underwent a coronary intervention.

### Blood sampling and laboratory methods

Blood was drawn directly upon admission. Routine laboratory parameters, including in-house troponin, C-reactive protein, creatinine, and creatinkinase-MB were measured immediately after blood withdrawal by standardized methods. Additionally, ethylenediaminetetraacetic acid plasma, citrate plasma, and serum samples were collected, centrifuged, and frozen at -80°C.

Investigational cTnI was determined using a commercial contemporary sensitive assay (TnI-Ultra, Siemens Healthcare Diagnostics, Germany) on an ADVIA Centaur XP system with measuring range of 6–50000 ng/L and lowest concentration with coefficient of variation of 10% or less at 30ng/L. The reference limit based on the 99^th^ percentile for a healthy population is 40ng/L [25].

B-type natriuretic peptide (BNP) was assayed on the ARCHITECT i System (Abbott Diagnostics, Germany) with analytical sensitivity of ≤10pg/ml and measuring range of 0–5000 pg/ml. Estimated glomerular filtration rate (eGFR) was calculated based on the abbreviated modification of diet in renal disease formula (MDRD)[27]. Based on availability of sample volume, BNP and cTnI levels were available in 417 and data on eGFR in 425 patients of 427 patients presenting with wide QRS complex.

### Statistical analyses

Variables with normal distribution were characterized by arithmetic mean and SD, whereas skewed variables were described by median and interquartile range. To evaluate possible correlations between some of the items correlation analyses were carried out using Spearman's rank correlation coefficient. Wilcoxon signed-rank sum test was used to compare continuous biomarker levels with skewed distribution between groups. To analyse the diagnostic performance of cTnI in patients with wide QRS complex and with LBBB, the respective area under the curve in receiver operator characteristic analyses (AUROC) was calculated. As proposed by DeLong et al., confidence intervals were constructed based on estimated respective covariance matrix[28]. Based on this AUROC data, different potential thresholds were established including an unweighted optimized diagnostic threshold by optimizing the Youden index as well as cut-offs with respect to a *rule-out* strategy associated with a sensitivity of 90% and to a *rule-in* strategy associated with specificity of 90%. These cut-offs were applied and the positive and negative predictive values (PPV and NPV) were calculated by applying the corresponding thresholds and consecutively calculating the corresponding values from a 2x2 table in the usual way, here 95% confidence limits for binomially distributed variables are given. The cut-offs were also applied in LBBB patients and the respective sensitivity, specificity, negative and positive predictive values were calculated.

Sensitivity, specificity, positive and negative predictive values of the Sgarbossa Algorithm was determined and compared to those of the cTnI cut-offs. Also sensitivity, specificity, positive and negative predictive values of the Sgarbossa algorithm in addition to the cTnI values were evaluated.

The diagnostic performance of the Sgarbossa index was compared to the cTnI cut-offs by comparing the respective Youden indices (using the net reclassification index as well as a new method based on publication of Chen et al. 2015)[29,30].

To make at least a tentative inference on the robustness of these estimated measures we created 200 bootstrap replicates of the dataset and report the average values and the 2.5% and 97.5% quantiles as edges of a nonparametric 95% confidence interval. All analyses were carried out using the R 3.1.1 software package (R Foundation for Statistical Computing, Vienna, Austria).

## Results

### Baseline characteristics

Of the 417 patients presenting with wide QRS complex, available investigational cTnI measurements and symptoms suggestive of an AMI, a significant obstructive CAD was documented in 117 patients who subsequently underwent a percutaneous coronary intervention or surgical revascularisation. Baseline characteristics of patients who underwent revascularisation as well as of the remaining 300 patients without a significant coronary stenosis are presented in Table 1. Most of the characteristics did not differ between the two patients groups. Of note, patients with a significant coronary stenosis were more likely diabetics (p = 0.02). Extent of CAD, quantified by the *SYNergy between PCI with TAXUSTM and Cardiac Surgery* (SYNTAX) score, was higher in the group who underwent revascularization with 15.5(8.0, 24.5) compared to 0(0, 6.8)(p<0.001) in patients without need of a coronary intervention.

**Table 1:** Baseline data.

	No	No Coronary Intervention Needed	Significant Coronary Stenosis	p-value
		n = 300	n = 117	
Age, yrs	417	65 (13)	65 (11)	0.74
Male sex	417	235/300 (78%)	100/117 (85%)	0.13
*Vital signs on admission*				
BP systolic, mmHg	414	144 (27)	145 (26)	0.91
BP diastolic, mmHg	414	78 (14)	79 (14)	0.44
Heart rate, BPM	412	73 (17)	73 (16)	0.67
*Cardiovascular Risk factors and history*				
Hypertension	417	248/300 (83%)	93/117 (79%)	0.54
Dyslipidemia	417	232/300 (77%)	93/117 (79%)	0.73
Obesity	379	87/270 (32%)	40/109 (37%)	0.47
Diabetes	417	61/300 (20%)	37/117 (32%)	0.02
Current smoker	417	50/300 (17%)	29/117 (25%)	0.08
Former smoker	401	100/288 (35%)	43/113 (38%)	0.61
Known CAD	417	154/300 (51%)	55/117 (47%)	0.49
Family CAD	416	89/299 (30%)	29/117 (25%)	0.37
History of MI	416	103/299 (34%)	39/117 (33%)	0.92
CHF	391	30/283 (11%)	10/108 (9%)	0.84
*Laboratory results on admission*				
Potassium, mmol/l	414	3.9 (3.7, 4.2)	3.9 (3.6, 4.1)	0.04
Creatinine, mg/dl	415	1.0 (0.8, 1.2)	1.0 (0.9, 1.1)	0.72
eGFR, mL/min	415	78.8 (60.4, 91.1)	79.6 (65.9, 89.5)	0.86
Creatinkinase U/l	415	102.5 (72, 157)	146 (93, 230.5)	< 0.001
C-reactive protein, mg/L	413	2.5 (1.3, 5.3)	2.8 (1.6, 6.7)	0.12
Total Cholesterol, mg/dl	367	188 (151, 223)	192 (160, 235.5)	0.11
HDL, mg/dl	367	46 (39, 58)	43 (36, 52)	0.02
LDL, mg/dl	365	106 (79.5, 142)	118.5 (92, 161)	0.01
Troponin I, ng/l	417	11 (4, 30)	250 (45, 1511)	< 0.001
BNP, pg/ml	408	51.9 (17.1, 164.5)	82.1 (22.1, 227.8)	0.07
*Home Medication*				
Aspirin	417	137/300 (46%)	52/117 (44%)	0.91
Clopidogrel	417	36/300 (12%)	16/117 (14%)	0.76
Statin	417	116/300 (39%)	44/117 (38%)	0.93
ARB	417	65/300 (22%)	20/117 (17%)	0.37
ACEi	417	122/300 (1%)	52/117 (44%)	0.55
ß-Blocker	417	146/300 (49%)	50/117 (43%)	0.33
Calcium Channel Blocker	417	47/300 (16%)	28/117 (24%)	0.07
Digitalis	417	24/300 (8%)	1/117 (1%)	0.01
Loop diuretics	417	67/300 (22%)	13/117 (11%)	0.01
Aldosterone Inhibitor	417	22/300 (7%)	3/117 (3%)	0.11
Oral Anticoagulants	417	37/300 (12%)	6/117 (5%)	0.05

Baseline characteristics of 417 patients presenting with acute chest pain or equivalent symptoms and wide QRS complex to a chest pain unit. Patients have been stratified according to whether they underwent a coronary intervention. Data presented as cases/number (percentage), mean (standard deviation) or median (interquartile range) as appropriate. Abbreviations: ACEi, angiotensin converting enzyme inhibitor; ARB, angiotensin receptor blocker; BNP, brain natriuretic peptide; BP, blood pressure; BPM, beats per minute; BMI, body mass index; CAD, coronary artery disease; CHF, congestive heart failure; CK, creatine kinase; CRP, C-reactive protein, GFR, glomerular filtration rate; Obesity is defined as body mass index > 30; HDL, high-density lipoprotein; LDL, low-density lipoprotein; MI, myocardial infarction.

### Biomarker concentrations on admission

**Fig 1:**
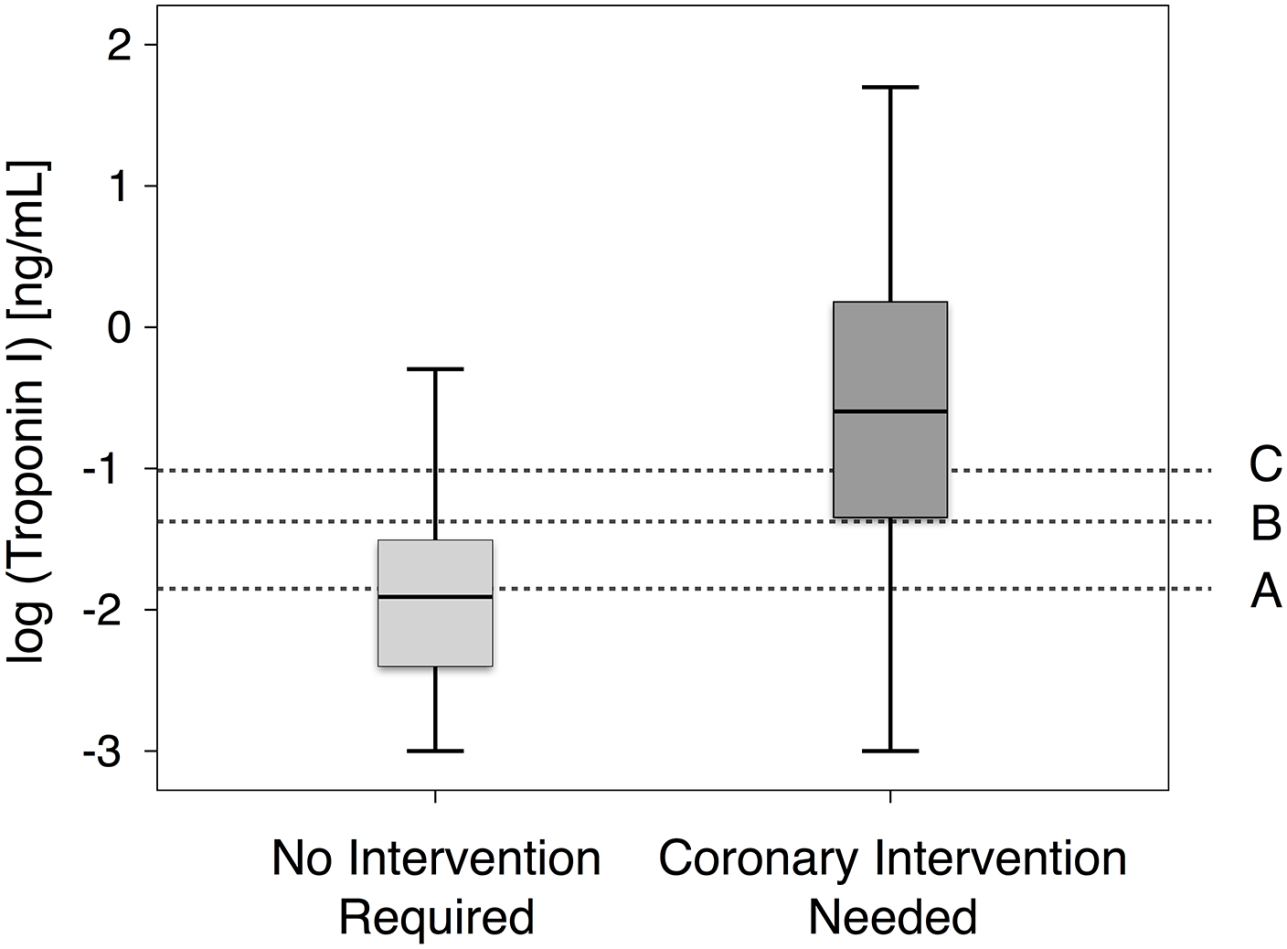
Troponin I at admission. Concentration of troponin I determined upon presentation in patients with acute chest pain and wide QRS complex in respect to presence of an acute coronary syndrome with need for coronary intervention compared to patients not needing a coronary intervention. Troponin I was available in 417 patients, data is presented log-transformed. Lines represent different troponin I thresholds associated with 90% sensitivity (A; 14 ng/L); 90% specificity (C; 96 ng/L) or unweighted with highest sum of sensitivity and specificity (B; 41 ng/L) for discrimination of patients needing a coronary intervention.

In patients presenting with suspected AMI and wide QRS complex, those with a significant coronary stenosis showed significantly higher median cTnI levels directly upon admission with 250ng/L compared to 11ng/L (p<0.001) in patients without significant stenosis (Fig 1).

As expected, patients with wide QRS complex had elevated BNP levels on admission. There was only a small difference between patients with and without significant coronary obstruction which was not statistically significant (median 82.1pg/ml vs. 51.9pg/ml p = 0.07).

BNP and cTnI levels determined on admission showed a significant correlation (r = 0.456, p<0.01). Likewise, a weaker but still significant correlation was observed between cTnI and eGFR (r = -0.220, p<0.01) and cTnI and age (r = 0.186, p<0.01).

### Identification of patients with significant coronary stenosis

**Fig 2:**
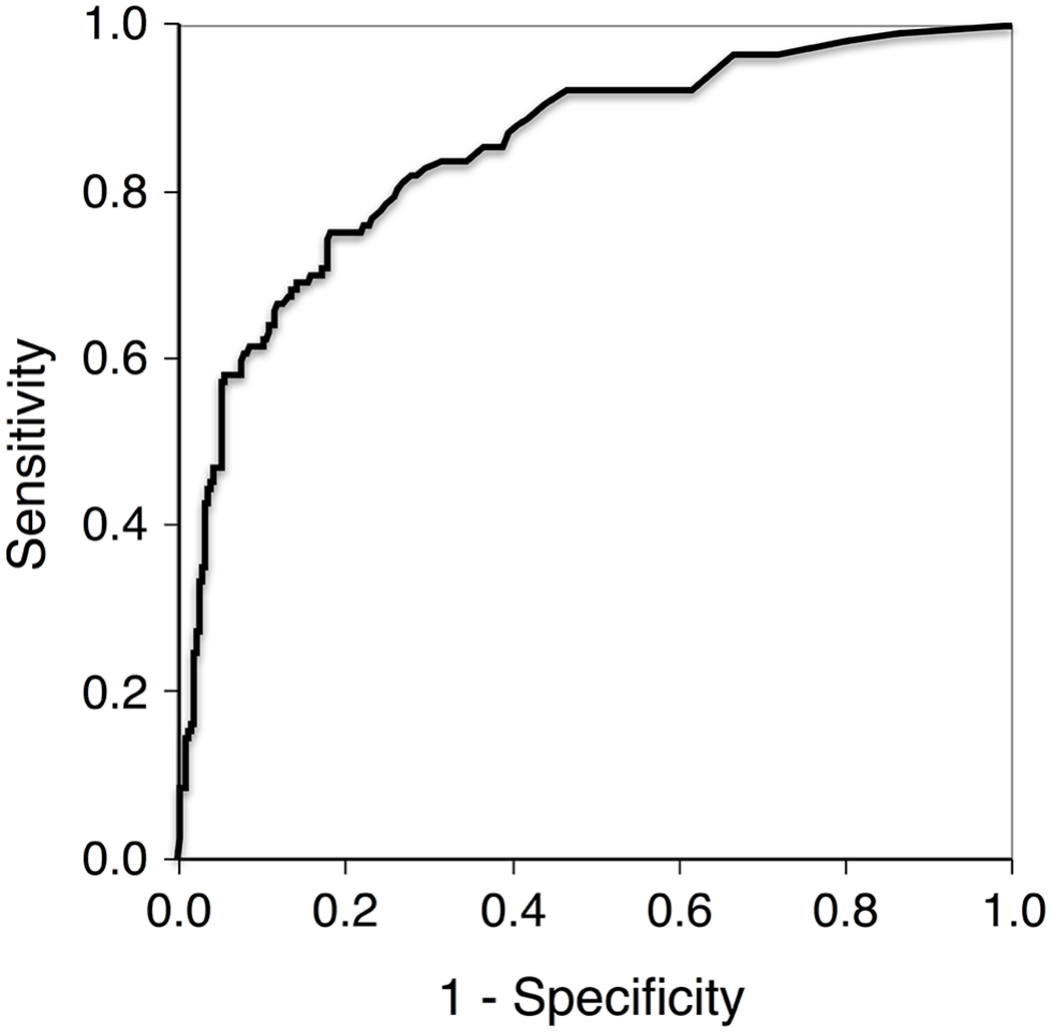
ROC curve for identification significant coronary stenosis. Receiver operating characteristic curves in patients with acute chest pain and wide QRS complex for identification of individuals needing coronary intervention by troponin I values determined upon admission. Area under the curve for troponin I is calculated with 0.849 (0.807–0.892) and Youden index with 0.56 (0.50–0.67).

cTnI determined on admission in hemodynamically stable patients presenting with symptoms suggestive of an AMI and wide QRS complex yielded an area under the curve (AUC) in the receiver operator characteristics analysis of 0.849 (0.807–0.892) to identify an underlying significant CAD (Fig 2). This value is lower than the AUC of the same cTnI assay in the unselected cohort irrespective of QRS duration to diagnose AMI with 0.96 as published[25]. Of note, this AUC refers to another diagnostic setting (AMI vs. significant CAD). Of interest, BNP as comparator, determined on admission, yielded an AUC of only 0.553 (0.492, 0.613) in this context. If restricting the analyses to patients with QRS duration of at least 120ms, cTnI yields an AUC of 0.805 (0.741, 0.869) to identify an underlying significant CAD. If considering only patients with prevalent LBBB, the respective AUC was 0.788 (0.715, 0.861).

**Fig 3:**
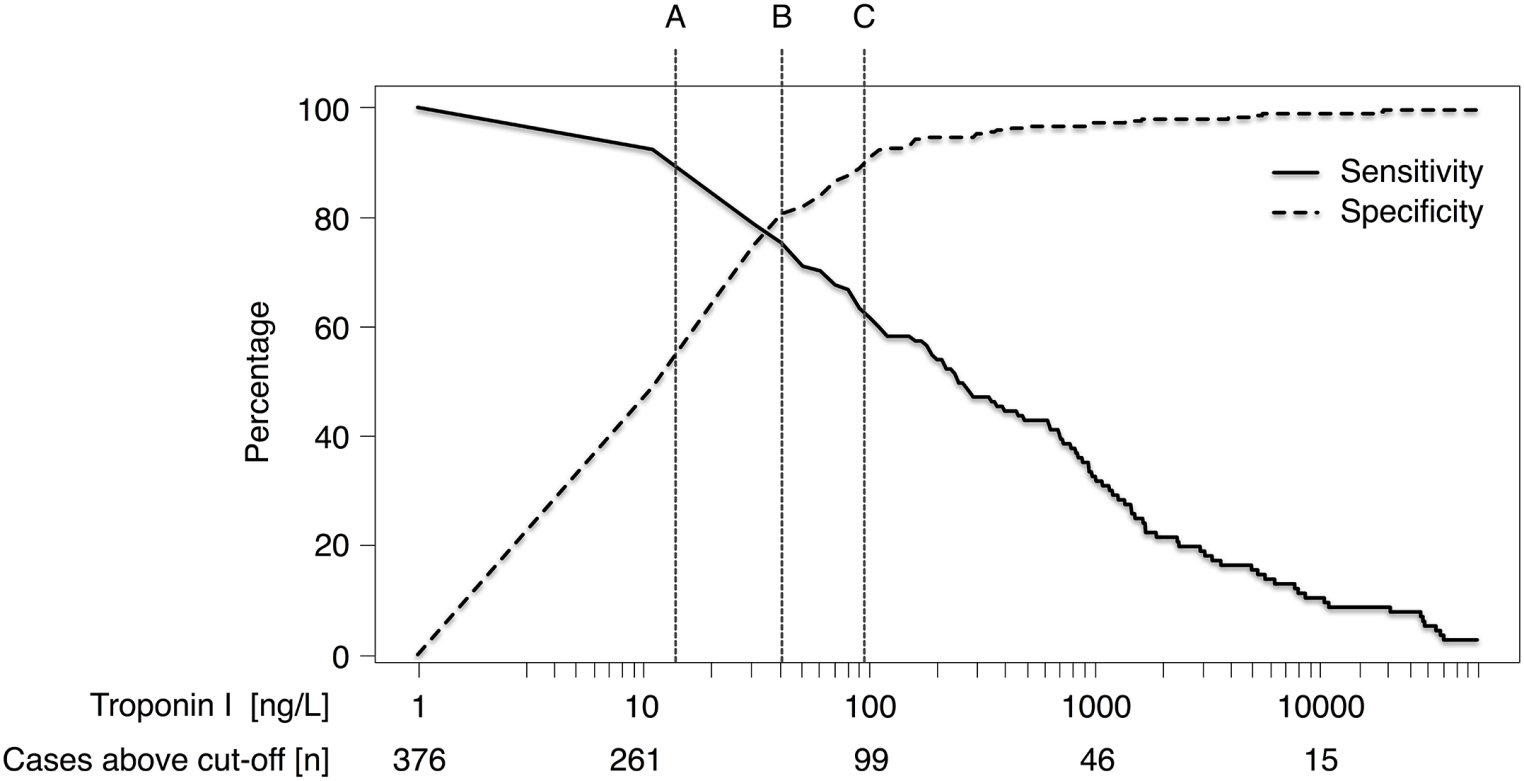
Sensitivity and Specificity of troponin I to identify individuals needing a coronary intervention. Sensitivity and Specificity of troponin I determined upon admission in patients with suspected acute coronary syndrome and wide QRS complex to identify individuals needing a coronary intervention. X-axis is presented in logarithmic scale. Lines represent different troponin I thresholds associated with 90% sensitivity (A; 14 ng/L); 90% specificity (C; 96 ng/L) or with highest sum of sensitivity and specificity (B; 41 ng/L).

To identify high-risk patients requiring coronary intervention in patients with wide QRS complex, the continuous sensitivities and specificities as a function of all possible cTnI threshold levels are depicted in Fig 3. As anticipated, specificity increases, whereas sensitivity declines with higher cTnI level.

### Derivation and application of different diagnostic cTnI cut-offs

With respect to clinical decision-making, we furthermore derived three diagnostic cTnI thresholds according to different evaluation strategies based on the sensitivities and specificities as shown in Fig 3.

First, to reflect a diagnostic *rule-out* approach, we calculated a specific cTnI cut-off concentration associated with a sensitivity as close as possible to 90% (sensitivity = 0.91) with 14ng/L.

Second, to reflect a diagnostic *rule-in* approach, we derived a cTnI cut-off concentration of 96ng/L that was associated with a specificity of 90%.

As third diagnostic threshold, an unweighted cut-off, was calculated with the highest sum of sensitivity and specificity with a concentration of 41 ng/L, quite close to the proposed 99^th^ percentile cut-off concentration of 40 ng/L of the respective cTnI assay. Fig 1 depicts, that such an unweighted threshold indeed distinguishes patients, who underwent a coronary intervention, quite well from those without a significant coronary stenosis.

Application of the proposed three optimized thresholds to identify patients requiring a coronary intervention with the respective NPV and PPV is presented in Table 2. Use of the sensitivity optimized cut-off yields a NPV of 94% (89%, 97%) compared to 89% (85%, 93%), if applying the unweighted or the 99^th^ percentile cut-off. With respect to a *rule-in* approach, the specificity optimized cut-off improved the PPV to 70% (60%, 79%) close to the 76.7% (72.7%, 80.4%) yielded by the application of the 99^th^ percentile cut-off in the overall cohort irrespective of QRS duration to identify AMI, as published earlier [25]. To validate these results, the three cut-offs were additionally applied to bootstrap replicates of the original cohort and here, these thresholds yielded comparable diagnostic information (S1 Table).

**Table 2:** Diagnostic performance of troponin I for identification of patients needing coronary intervention.

Troponin ICut-off Values	Sensitivity	Specificity	PPV	NPV
14 ng/L	0.91 (0.84–0.95)	0.56 (0.5–0.62)	0.45 (0.38–0.51)	0.94 (0.89–0.97)
41 ng/L	0.75 (0.66–0.83)	0.81 (0.76–0.85)	0.6 (0.52–0.68)	0.89 (0.85–0.93)
96 ng/L	0.62 (0.52–0.7)	0.9 (0.86–0.93)	0.7 (0.6–0.79)	0.86 (0.81–0.89)

Diagnostic performance with sensitivity, specificity, positive predictive and negative predictive value (PPV, NPV) of cardiac troponin I determined upon presentation for identification of patients needing coronary intervention in individuals presenting with suspected acute coronary syndrome and wide QRS complex. Cut-offs were derived to be as close as possible to 90% sensitivity (14 ng/L) to reflect a diagnostic rule-out approach, to 90% specificity (96 ng/L) in respect to rule-in or unweighted with highest sum of sensitivity and specificity (41 ng/L).

We also applied the respective optimized cut-offs in LBBB patients. The respective sensitivitiy, specificity, positive and negative predictive value of LBBB patients was comparable to those in the overall cohort; detailed information is presented in the supporting information (S2 Table).

Of note, in 187 patients (44.8%), cTnI levels were less or equal to 14ng/L L (*rule-out* approach) and in 103 patients (24.7%) greater or equal to 96ng/L (*rule-in* approach) with potential impact on diagnostic decision making, leaving under one third of the evaluated individuals (n = 127, 30.5%) in a so-called troponin observation zone, in whom serial troponin testing is mandatory. Regarding potential safety concerns, of those 187 individuals with cTnI below the *rule-out* cut-off, 13 patients required subsequent coronary revascularisation. None of those patients in the *rule-out* zone died or developed an AMI after the initial hospitalization within a 6-month follow-up period.

### Sgarbossa algorithm

By applying the Sgarbossa algorithm, an Sgarbossa index ≥ 3 yield a specificity of 87% (81–92%) and therefore can be seen as a good predictor of a significant coronary stenosis with respect to a *rule-in* approach. Adding the specificity optimized cTnI cut of, further specificity can be gained (94% (89–97%)).

In contrast with respect to a *rule-out* approach, the Sgarbossa index showed just a poor sensitivity by 34% (21%-49%) which could not be improved by adding the sensitivity optimized cut-off; detailed information is presented in the supporting information (S3 Table).

Using the net reclassification index, the Sgarbossa index showed a lower Youden index compared to the optimized cut-offs but without statistical significance (cTnI cut-offs compared to Sgarbossa index with respective p-value: cTnI 14ng/L p = 0.1692, cTnI 41ng/L p = 0.231 and cTnI 96ng/L p = 0.1719).

We also applied a new method to compare Youden indices. By applying this method, the Sgarbossa index yielded statistically significant lower Youden indices (cTnI cut-offs compared to Sgarbossa index with respective p-values 14ng/L p = 0.02, cTnI 41ng/L p = 0.008 and cTnI 96ng/L p = 0.039)[29,30].

## Discussion

Diagnosis of a significant coronary stenosis or an AMI as a STEMI equivalent in the presence of a wide QRS complex and suspected LBBB can be quite difficult, as many common ECG criteria cannot be used in this context. Due to the diagnostic uncertainty in the ER, the question often remains which patient actually might benefit from an early coronary intervention. The aim of the present study was to support the decision of the treating physician with an additional diagnostic instrument. We evaluated the use of cTnI determined directly upon admission in hemodynamically stable patients presenting with a wide QRS complex and symptoms suggestive of an AMI to help identifying those patients, who do have a significant coronary stenosis and may benefit from an early aggressive and invasive treatment strategy.

The results of our study demonstrate, that, first, cTnI levels of patients presenting with suspected AMI, wide QRS complex and angiographically confirmed coronary stenosis are significantly higher than cTnI levels of patients without significant coronary stenosis;

Second, cTnI levels of at least 96 ng/L, a threshold derived to optimize specificity, are associated with a significantly improved positive predictive value to *rule-in* patients, who show a significant coronary lesion and may profit from an early coronary intervention;

Third, *rule-out* of patients who do not need a coronary angiography is facilitated using a sensitivity optimized cut-off of 14 ng/L or less; and finally, only 30.5% of our patients presented with cTnI levels between 14 ng/L and 96 ng/L comprising a "grey area", in which neither an early *rule-in* nor *rule-out* is possible based on cTnI determination. These patients might therefore require further observation and diagnostic work-up including serial biomarker determination.

Current recommendations suggest to treat patients with supposedly new LBBB as STEMI equivalent [2]. Additionally, development of an RBBB in the setting of an acute coronary syndrome has been associated with unfavourable prognosis [23]. Therefore a robust diagnostic *rule-in* is crucial for early risk stratification and decision making with respect to different treatment strategies. The results of the present study demonstrate that a specificity optimized cTnI threshold yielded a PPV of 70% (60%, 79%), in a comparable magnitude of the PPV of 76.7% (72.7%, 80.4%) of the same cTnI assay determined in unselected early presenting chest pain patients to identify individuals suffering an AMI. This underlines the feasibility to use cTnI in hemodynamically stable patients to identify those individuals at risk as recommended by the guidelines.

With respect to the high number of patients presenting with possibly new LBBB of up to 70% (16) not suffering an underlying acute significant coronary obstruction, the concept of applying a sensitivity optimized cut-off for rapid *rule-out* achieved a high NPV of 94% (89%, 97%), which is comparable to cTnI measurement in unselected chest pain patients with NPV of 96.4% (95.2%, 97.5%)(25) to exclude AMI.

Finally, the application of such *rule-in* and *rule-out* optimized thresholds leads to a large proportion of patients (69.5%), in whom an early decision could be made such as timely coronary angiography or further non-coronary focused diagnostic work-up. This leaves only about one third of patients, who should stay in a chest pain observation unit for further evaluation with respective impact on logistics and economics. Next desirable step would be, in our opinion, to test this concept with the proposed thresholds in an independent prospective cohort.

### Limitations

The present study focused on patients presenting with a wide QRS complex defined by a QRS duration of at least 110ms. The common ECG criteria (including, e.g. the Sgarbossa criteria) refer to LBBB[6] rather than RBBB. Furthermore, according to the current guidelines, only LBBB is seen as a STEMI equivalent[2]. Still, data on the sub-group of patients with prevalent LBBB showed comparable results. Moreover, unfortunately, no complete data on previously known LBBB was available, therefore all patients were defined as presenting with potentially new QRS prolongation. Furthermore, as not all evaluated patients underwent coronary angiography this has to be seen as limitation, potentially introducing a bias. However the studied cohort is based on an all-comers real world observational study, hence such a desirable diagnostic homogeneity cannot be achieved and might not represent daily clinical routine.

The need for a coronary intervention or bypass surgery was regarded as a relevant coronary stenosis and used as the endpoint of the present study. The indication to coronary intervention was based on the decision of the treating physician only on the basis of visual criteria. No technical support such as optical coherence tomography (OCT) was used. The decision of the treating physician might therefore be subjective. [31]

## Conclusion

The result of the present study demonstrates that, in hemodynamically stable patients presenting with acute chest pain and wide QRS complex, cTnI levels assayed sensitively directly upon admission are a helpful additional diagnostic instrument. In these patients, the use of sensitivity or specificity optimized diagnostic cTnI thresholds improves timely *rule-in* and *rule-out* with respect to the presence of a significant coronary lesion and therefore the need for an early coronary angiography.

## Supporting Information

S1 Table Validation of the diagnostic performance in 200 bootstrap replicates of the cohort. Shown are sensitivity, specificity, positive predictive and negative predictive value (PPV, NPV) of troponin I determined upon presentation for identification of patients needing coronary intervention in a validation cohort based on the initial cohort including individuals presenting with suspected acute coronary syndrome and wide QRS complex. Data presented as percentage with corresponding 95% confidence interval. Abbreviations: NPV (negativ predictive value); PPV (positive predictive value).
(DOCX)

S2 Table Diagnostic performance of troponin I in the presence of LBBB for identification of patients needing coronary intervention. Diagnostic performance with sensitivity, specificity, positive predictive and negative predictive value (PPV, NPV) of cardiac troponin I determined upon presentation for identification of patients needing coronary intervention in individuals presenting with suspected acute coronary syndrome and left bundle branch block (LBBB). Cut-offs were derived in the overall cohort in patients with wide QRS complex and suspected acute coronary syndrome.
(DOCX)

S3 Table Diagnostic performance of Sgarbossa algorithm as well as troponin I in addition to Sgarbossa algorithm for identification of patients needing coronary intervention. Diagnostic performance with sensitivity, specificity, positive predictive and negative predictive value (PPV, NPV) of Sgarbossa algorithm according to ECG criteria on admission as well as Troponin I cut offs in addition to Sgarbossa algorithm for identification of patients needing coronary intervention in individuals presenting with suspected acute coronary syndrome and wide QRS complex. Cut-offs were derived to be as close as possible to 90% sensitivity (14 ng/L) to reflect a diagnostic rule-out approach, to 90% specificity (96 ng/L) in respect to rule-in or unweighted with highest sum of sensitivity and specificity (41 ng/L) in the overall cohort.
(DOCX)
