# Supplementary material for: Troponin I Assay for Identification of a Significant Coronary Stenosis in Patients with Suspected Acute Myocardial Infarction and Wide QRS Complex
Source: PLoS One. 2016 May 5;11(5):e0154724. doi: 10.1371/journal.pone.0154724 (PMC4858235; doi:10.1371/journal.pone.0154724)
Supplement: S1 Table — Shown are sensitivity, specificity, positive predictive and negative predictive value (PPV, NPV) of troponin I determined upon presentation for identification of patients needing coronary intervention in a validation cohort based on the initial cohort including individuals presenting with suspected acute coronary syndrome and wide QRS complex. Data presented as percentage with corresponding 95% confidence interval. Abbreviations: NPV (negativ predictive value); PPV (positive predictive value). (DOCX) [file pone.0154724.s001.docx]

| **Troponin I**  **Cut-off Values** | **Sensitivity** | **Specificity** | **PPV** | **NPV** |
| --- | --- | --- | --- | --- |
| 14 ng/L | 0.91 (0.85-0.97) | 0.56 (0.50-0.61) | 0.44 (0.38-0.50) | 0.94 (0.90-0.98) |
| 41 ng/L | 0.75 (0.65-0.83) | 0.81 (0.76-0.85) | 0.61 (0.52-0.69) | 0.89 (0.86-0.93) |
| 96 ng/L | 0.61 (0.53-0.69) | 0.90 (0.86-0.93) | 0.70 (0.60-0.78) | 0.86 (0.82-0.89) |
