# Supplementary material for: Troponin I Assay for Identification of a Significant Coronary Stenosis in Patients with Suspected Acute Myocardial Infarction and Wide QRS Complex
Source: PLoS One. 2016 May 5;11(5):e0154724. doi: 10.1371/journal.pone.0154724 (PMC4858235; doi:10.1371/journal.pone.0154724)
Supplement: S2 Table — Diagnostic performance with sensitivity, specificity, positive predictive and negative predictive value (PPV, NPV) of cardiac troponin I determined upon presentation for identification of patients needing coronary intervention in individuals presenting with suspected acute coronary syndrome and left bundle branch block (LBBB). Cut-offs were derived in the overall cohort in patients with wide QRS complex and suspected acute coronary syndrome. (DOCX) [file pone.0154724.s002.docx]

| **Troponin I**  **Cut-off Values**  **in LBBB**  **patients** | **Sensitivity** | **Specificity** | **PPV** | **NPV** |
| --- | --- | --- | --- | --- |
| 14 ng/L | 0.88 (0.76-0.94) | 0.45 (0.36-0.53) | 0.39 (0.30-0.48) | 0.90 (0.80-0.96) |
| 41 ng/L | 0.72 (0.59-0.83) | 0.71 (0.63-0.78) | 0.5 (0.39-0.61) | 0.87 (0.79-0.92) |
| 96 ng/L | 0.55 (0.42-0.68) | 0.86 (0.79-0.91) | 0.60 (0.46-0.73) | 0.83 (0.76-0.88) |
