# Supplementary material for: Troponin I Assay for Identification of a Significant Coronary Stenosis in Patients with Suspected Acute Myocardial Infarction and Wide QRS Complex
Source: PLoS One. 2016 May 5;11(5):e0154724. doi: 10.1371/journal.pone.0154724 (PMC4858235; doi:10.1371/journal.pone.0154724)
Supplement: S3 Table — Diagnostic performance with sensitivity, specificity, positive predictive and negative predictive value (PPV, NPV) of Sgarbossa algorithm according to ECG criteria on admission as well as Troponin I cut offs in addition to Sgarbossa algorithm for identification of patients needing coronary intervention in individuals presenting with suspected acute coronary syndrome and wide QRS complex. Cut-offs were derived to be as close as possible to 90% sensitivity (14 ng/L) to reflect a diagnostic rule-out approach, to 90% specificity (96 ng/L) in respect to rule-in or unweighted with highest sum of sensitivity and specificity (41 ng/L) in the overall cohort. (DOCX) [file pone.0154724.s003.docx]

| **Troponin I**  **Cut-off Values** | **Sensitivity** | **Specificity** | **PPV** | **NPV** |
| --- | --- | --- | --- | --- |
| Index ≥ 3 | 0.34 (0.21, 0.49) | 0.87 (0.81, 0.92) | 0.43 (0.27, 0.61) | 0.82 (0.75, 0.87) |
| Index ≥ 3 and  Troponin ≥ 14ng/L | 0.34 (0.21, 0.49) | 0.89 (0.83-0.93) | 0.48 (0.31-0.66) | 0.81 (0.75-0.87) |
| Index ≥ 3 and  Troponin ≥ 41ng/L | 0.28 (0.16, 0.43) | 0.93 (0.88-0.96) | 0.54 (0.33-0.74) | 0.81 (0.74-0.86) |
| Index ≥ 3 and  Troponin ≥ 96ng/L | 0.17 (0.08-0.31) | 0.94 (0.89-0.97) | 0.47 (0.23-0.72) | 0.79 (0.72-0.84) |
